# Epigenetic Function of Activation-Induced Cytidine Deaminase and Its Link to Lymphomagenesis

**DOI:** 10.3389/fimmu.2014.00642

**Published:** 2014-12-18

**Authors:** Pilar M. Dominguez, Rita Shaknovich

**Affiliations:** ^1^Division of Hematology and Oncology, Weill Cornell Medical College, New York, NY, USA; ^2^Department of Pathology and Laboratory Medicine, Weill Cornell Medical College, New York, NY, USA

**Keywords:** activation-induced cytidine deaminase, DNA methylation, epigenetics, B cells, lymphomagenesis

## Abstract

Activation-induced cytidine deaminase (AID) is essential for somatic hypermutation and class switch recombination of immunoglobulin (Ig) genes during B cell maturation and immune response. Expression of AID is tightly regulated due to its mutagenic and recombinogenic potential, which is known to target not only Ig genes, but also non-Ig genes, contributing to lymphomagenesis. In recent years, a new epigenetic function of AID and its link to DNA demethylation came to light in several developmental systems. In this review, we summarize existing evidence linking deamination of unmodified and modified cytidine by AID to base-excision repair and mismatch repair machinery resulting in passive or active removal of DNA methylation mark, with the focus on B cell biology. We also discuss potential contribution of AID-dependent DNA hypomethylation to lymphomagenesis.

## Dynamic Nature of Methylome during B Cell Development

Epigenetic mechanisms of regulation including histone modifications, non-coding RNA-mediated gene regulation, chromatin remodeling, and DNA methylation (1) play an important role in B cell differentiation and the antibody response (2). DNA methylation is essential during X-chromosome inactivation, imprinting, and tissue differentiation (3, 4). This epigenetic modification refers to the addition of a methyl group at the C5 position of cytosine (C), mostly when C is bound to guanine (G) creating a CpG site in mammalian organisms (5, 6), with less common non-CpG methylation detected in embryonic stem cells (ESCs) and brain tissue (7, 8). The addition of the methyl group is catalyzed by the family of DNA methyltransferases (DNMTs) (9), whereas DNA demethylation can be a passive or an active process. Passive demethylation occurs when methylC (mC) mark is not faithfully reproduced either during replication or due to DNA damage, while active demethylation requires the action of enzymes and can be replication-independent (10, 11). However, the molecules involved in the 5-methylC (5mC) active demethylation are still not clearly defined (see below). Several studies have investigated the DNA methylation landscape in B cells and the role of the DNA methylome in B cell development. Ji et al. demonstrated in a mouse model using Comprehensive High-throughput Array-based Relative Methylation analysis (CHARM) (12) that lymphoid commitment requires more DNA methylation than myeloid lineage with myeloid skewing of lineages in DNMT1 hypomorphic animals. Loss of methylation predominates during progression of Multipotent Progenitors (MPPs) to Common Lymphoid Progenitors (CLPs) (13, 14). During transition from naïve B cells (NBs) to germinal center B cells (GCBs), occurring in the secondary lymphoid organs after T cell-mediated activation, there is marked demethylation of the genome (15, 16). Memory and plasma cells display DNA methylation patterning very similar to GCBs, although the three subpopulations of B cells differ substantially at the transcriptional level (16). The use of the DNA demethylating agent Decitabine results in complete abrogation of the GCs, while preserving the primary follicles (15), underscoring an important biological role of methylation in B cell development. The fact that entrance of NBs into the GC reaction is characterized by marked upregulation of DNMT1 and simultaneous hypomethylation of many genomic loci suggests yet unknown mechanisms of passive or active demethylation after B cell activation. In this review, we would like to explore the potential role of activation-induced cytidine deaminase (AID) in modifying the methylome of mature B cells. We will present and analyze large amount of conflicting evidence that accumulated up to date linking AID with epigenetic modifications during development and B cell differentiation.

## Function of AID in B Cell Development

NBs enter the GC reaction and undergo marked changes in transcriptional program, including dramatic upregulation of the enzyme AID (or AICDA), a member of the APOBEC family of cytidine deaminases that is required for both somatic hypermutation (SHM) and class switch recombination (CSR) (17–19). SHM results in the introduction of somatic mutations in the rearranged V(D)J region of the Ig genes (IgV) in order to generate antibodies with high affinity antigen binding sites (20, 21). Additionally, GCBs undergo a process of CSR of the constant region of the immunoglobulin (Ig) heavy chain (C_H_), generating isotypes with different immunological functions but the same antigen-specificity (22–24). Both SHM and CSR are initiated by the deaminase activity of AID, which is able to convert deoxycytosines (dC) into deoxyuracils (dU) in a single-stranded DNA, producing dU:dG mismatches that are removed by base-excision repair (BER) and mismatch repair (MMR) pathways (19, 25, 26). AID can also deaminate 5mC to thymine (T), although with less efficiency (27). AID is expressed in B cells in a stage specific manner during transition of NB through the GCs (28). NBs integrate signals through B cell receptor (BCR) and Toll-like receptors (TLRs), along with stimulation via surface receptors, such as CD40 and cytokine receptors, to initiate the NF-κB-mediated AID transcription (29). In the secondary lymphoid organs, AID is expressed in a subpopulation of dark and outer zone GCBs and in large extrafollicular B cells, which have evidence of CD40 and BCR stimulation (28), and is downregulated after differentiation of GCBs to memory and/or plasma cells (30, 31). In addition, viral infection of B cells can induce expression of AID (31–34). Ig genes are the main targets of AID, with a mutation rate of 10^−4^ to 10^−3^/bp per generation (35, 36). Nonetheless, non-Ig genes such as *BCL6, CD79A, CD79B*, or *CD95* are also susceptible to AID-mediated mutations (37–40). Moreover, Liu et al. reported that around 25% of the highly expressed genes in GCBs, including *Bcl6, Cd83, Pim1, Pax5*, and *Myc* among others, experienced AID-mediated deamination and low-level of SHM (41). The authors demonstrated that these non-Ig genes were protected from mutations in normal B cells due to the activity of high-fidelity BER factors. Besides, they observed that there was correlation between target regions of AID and sites of chromosomal translocations and deletions present in human lymphomas (41). They proposed that malfunction of repair machinery could lead to AID-mediated mutations and chromosomal instability, and finally to tumorigenesis. In line with these results, Yamane et al. performed chromatin immunoprecipitation sequencing (CHIP-seq) for AID in *ex vivo* activated B cells and proposed that AID was recruited genome-wide by stalled PolII polymerases, inducing low-level of hypermutation in those AID-targeted genes (42). Importantly, these findings provided insight into the role of AID in lymphomagenesis.

## Evidence Linking AID to DNA Demethylation

There is a body of accumulating evidence linking AID to genome-wide epigenetic changes, and specifically to DNA demethylation (43). A significant discovery has been made in recent years that implicated AID in DNA demethylation in three paradigms: epigenetic reprogramming in heterokaryons using mouse ESCs, demethylation in zebrafish, and global demethylation in mouse primordial germ cells (PGCs) (44–46). The capacity of AID to deaminate 5mC in a single-stranded DNA was established *in vitro* (27), although the efficiency of 5mC deamination by AID was 5–10 times lower compared to unmethylated-C (47). The same report by Morgan et al. (27) demonstrated that AID mRNA was highly expressed in oocytes and ovaries, and moderately expressed in pluripotent cells (embryonic germ cells, ESCs, and PGCs), which can undergo epigenetic reprograming. Those results indicated that expression of AID was not restricted to activated B cells in the GCs of lymphoid organs and prompted the authors to suggest that the upregulation of AID (and other members of the APOBEC family) in pluripotent tissues could play a role in the epigenetic reprogramming during development. Rai et al. followed that hypothesis and presented the first evidence of the epigenetic role of AID (45). They proposed an active DNA demethylation mechanism in zebrafish embryos in which 5mC was converted to T through the cytosine deaminase activity of AID and the subsequent G:T mismatch was repaired by the thymine glycosylase methyl-CpG binding domain protein 4 (MBD4). The injection of methylated DNA (M-DNA) at the single-cell stage induced genome-wide demethylation in zebrafish embryos, allowing the analysis of epigenetic changes in both the injected M-DNA and the bulk genome. After knockdown of AID/APOBEC enzymes using anti-sense morpholino-modified oligonucleotides there was a reduction in demethylation. On the contrary, overexpression of AID (or APOBEC2A/B) and MBD4 induced DNA demethylation of M-DNA and the embryo genome (45).

Thereafter, the production of interspecies heterokaryons (mouse ESCs fused to primary human fibroblasts using polyethylene glycol) allowed the identification of AID-mediated demethylation as a key process for nuclear reprogramming toward pluripotency (44). Transfection experiments with small interference RNAs (siRNAs) for mouse and human AID mRNA 24 h before fusion inhibited expression of the ESC-specific genes *OCT4* and *NANOG* and reduced the CpG demethylation in the promoter of those genes. The transient overexpression of the human AID protein before siRNA transfection returned *NANOG* promoter demethylation and gene expression to normal levels and partially rescued *OCT4* demethylation and gene expression.

Primordial germ cells also undergo a process of epigenetic reprogramming, including DNA demethylation, which is pivotal for the acquisition of pluripotency during the germ line development (48, 49). Popp et al. demonstrated, on a genome-wide scale, that the genome from fully reprogramed PGCs at E13.5 was extensively hypomethylated and that the absence of AID increased DNA methylation levels, mainly in introns and repetitive elements and also in exons, but not in the promoter regions (46). Importantly, the epigenetic effect of AID on the genome of PGCs was confirmed by two independent techniques: bisulfite next generation sequencing (BS-Seq) and Sequenom MassARRAY. This finding of DNA hypermethylation in cells from *Aicda*^−^*^/^*^−^mice was restricted to PGCs since the genome-wide methylation levels remained unaffected in the fetus, the placenta, and the sperm. A biological process required during embryogenesis is the epithelial–mesenchymal transition (EMT), in which epithelial cells acquire a mesenchymal phenotype characterized by increased migratory capacity and invasiveness and production of extracellular components (50). In addition, EMT is a driving force for tumor metastasis (51). AID is upregulated in epithelial cells during inflammation *in vivo* (52–54) and by inflammatory factors *in vitro* (55, 56) and was shown to be required for the EMT in both normal mammary epithelial cells and breast cancer cell lines (55). Knockdown of AID in epithelial cell lines blocked the upregulation of *SNAI2, ZEB1*, and *ZEB2*, which are master regulator genes for EMT. Those genes inhibited by AID-deficiency presented high levels of methylation in the CpG islands associated with the promoters and were re-expressed in the presence of the demethylating agent 5-aza-2′deoxycitidine, indicating that AID regulated the transcription of EMT-associated genes through DNA demethylation (55).

The *in vitro* generation of induced pluripotent stem cells (iPSCs) by Yamanaka’s group from adult somatic cells through the addition of four transcription factors: OCT4, KLF4, SOX2, and c-MYC (57) opened new possibilities for regenerative medicine and autologous therapies. AID was identified as a critical factor for the initiation of mouse embryonic fibroblasts (MEFs) reprogramming to iPSCs (58). Inhibition of AID expression with shRNAs during the first 72 h after reprogramming induction reduced the number of iPSCs colonies. In addition, rescue experiments with a construct containing a catalytically deficient version of AID (E58Q) demonstrated that the deaminase activity of AID was required for the induction of iPSCs (58). However, experiments performed by different groups with *Aicda*^−^*^/^*^−^ mice challenged this conclusion because AID-deficient fibroblasts were able to generate iPSCs, although with different kinetics depending on the concentration of virus supernatant, the transfection plasmids, and the culture conditions, including the number of cell passages (59–61). Interestingly, Kumar et al. observed that cells from *Aicda*^−^*^/^*^−^mice generated iPSCs, but failed to stabilize the expression of pluripotency genes after 3 weeks of culture (59), a phenomenon that correlated with genome hypermethylation in reprogramming cells, especially near RGYW motifs. Additionally, genes that were upregulated later during reprogramming (*Rex1, Gdf3, Dnmt3l, Dnmt1, Apobec1, Cbx7*, and *Zfp96*) presented higher levels of methylation, both by RRBS and Sequenom MassArray, and were not expressed in the absence of AID (59). The authors proposed that AID was essential for the late phase of reprogramming, which is characterized by the genome-wide erasure of DNA methylation to stabilize a pluripotent phenotype (62).

Although AID is highly expressed in the GCBs and is responsible for the generation of high affinity antibodies through the induction of SHM and CSR (20, 43), to date only few studies have addressed the epigenetic role of AID in activated B cells (15, 63). An important link between SHM in B cells and DNA hypomethylation has been made by several studies (19, 64, 65). Endonuclease sensitivity sites within the loci containing CpG nucleotides and located close to J-C intronic enhancers are methylated in somatic cells, but are demethylated in B cells (66–68). Jolly et al. studied DNA methylation and SHM in *Jκ5* region, due to the high density of CpGs and high level of SHMs in those loci. They demonstrated that the locus was heavily methylated in mouse tail DNA, whereas it was totally demethylated in the GCBs from Peyer’s patches (64). Studying the Igκ transgene and analyzing the *Jκ5* locus they were able to demonstrate that B cells contained a mixture of loci with different state of methylation. Most importantly, the degree of demethylation correlated with the burden of SHM: only demethylated loci contained detectable mutations. This finding led the authors to conclude that transcription and demethylation are requited for SHM, and thus for AID targeting. Our current view is that it is equally possible that demethylation is a consequence of the deaminase activity of AID, which introduces mutations that are subsequently repaired. Hypomethylation along with double strand DNA breaks (DSBs) are likely to be an unwanted consequence of SHM. It is not clear how efficient are DNMTs (DNMT1 and DNMT3b are expressed in the GCBs (15)] in remethylating aberrantly hypomethylated loci. There does not seem to be a detectable loss of 5mC in the GCBs based on high-performance liquid chromatography (HPLC) measurements in isolated B cell fractions. Nevertheless, the methylation profiling indicates that there is a locus-specific loss of methylation in GCBs (15). DNMT1 localizes to the sites of DSBs and phosphoH2AX foci, and its absence results in increased number of DSBs, making it a likely candidate to remethylate AID-dependent demethylated loci (69). Global post-replicative remethylation of DNA depends on association of DNMT1 with PCNA and replication machinery. In addition, DNMT1 has been shown to associate with various transcription factors and remethylate various genes during not only S phase, but also G1 and G2 phases of the cell cycle (70, 71). Hervouet et al. also demonstrated that some genes remained hemi-methylated when leaving S phase and showed a delay in remethylation later in the cell cycle (70). How cell cycle and proliferative rate of GCBs affect global and locus-specific demethylation is an interesting question. It is conceivable that faster proliferation with shorter cell cycle may result in accumulation of hypomethylation. The extent of such passive demethylation is likely to be limited due to the protective effect of Hayflick limit of cell divisions in normal cells (72, 73). In cancer, on the other hand, this stochastic accumulation of hypomethylation may contribute to detectable levels of genomic hypomethylation. However, our data in diffuse large B cell lymphoma (DLBCL) cell lines does not reveal different degrees of genome methylation in cell lines with different duration of the cell cycle (unpublished data).

Using microarray-based DNA methylation profiling of NBs and GCBs (15), we demonstrated that hypomethylated loci within GCBs were significantly associated with RGYW-like AID-recognition motif (74) and CHIP-seq experiments identified AID-binding sites (42), providing another line of indirect evidence for the link between AID and DNA hypomethylation in B cells. On the other hand, Fritz et al. performed *ex vivo* experiments using *Aicda*^−^*^/^*^−^mice and reported that the absence of AID did not affect the methylome of activated B cells generated from splenic CD43^−^B cells stimulated with lipopolysaccharide (LPS), interleukin-(IL)-4, and anti-CD40. However, the authors did not exclude that AID could function as a DNA demethylase *in vivo* (63). Indeed, this *ex vivo* system of mouse B cell activation is not equivalent to *in vivo* GC reaction. For instance, the mutation rate in activated B cells is one order of magnitude lower than in GCBs (41, 75, 76). In addition, Hogenbirk et al. profiled purified GCB from immunized mice using MethylCap-Seq and failed to detect any difference in DNA methylation between wt and *Aicda*^−^*^/^*^−^mice (77). Nevertheless, it would be necessary to apply higher resolution genome-wide techniques to definitely answer this question. A useful tool to prove the demethylase function of AID in B cells would be a mouse model of GC-specific overexpression of AID, since to date the only available conditional AID-transgenic mice overexpress AID under the CD19 promoter, a molecule expressed in B cells from the early stages of differentiation (78). Therefore, better tools and approaches are necessary to elucidate the role of AID in the DNA demethylation process that occurs in B cells during their transit through the GC reaction (15).

## Proposed Mechanisms of Active Demethylation

**Figure 1:**
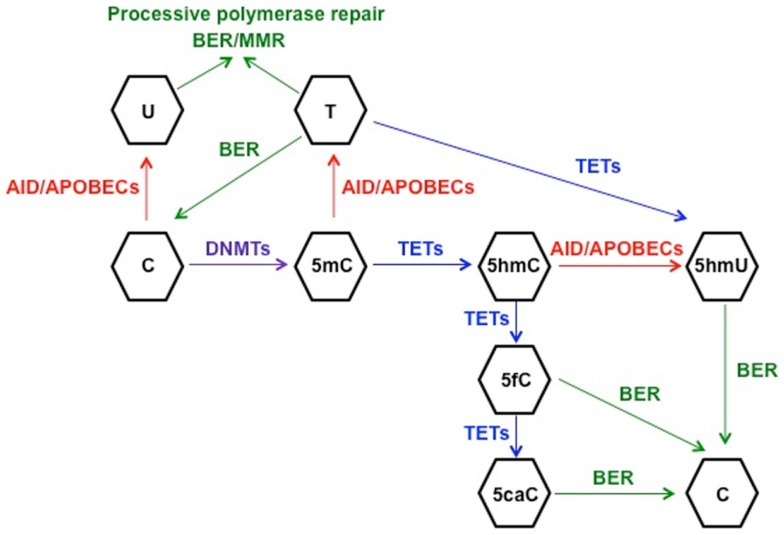
**Schematic representation of the proposed mechanisms for active DNA demethylation**. DNMTs catalyze the methylation of cytosine (C) to 5-methyl-C (5mC), which can be deaminated to thymine (T) by AID. The generated mismatch can be repaired by the short-patch BER machinery, restoring C, or by one of the processive repair pathways (long-patch BER or MMR), leading to demethylation of a fragment of DNA. This machinery involving processive DNA polymerases can also repair the uracil:guanine (dU:dG) mismatches generated after C deamination to U. On the other hand, TET-mediated hydroxylation of 5mC generates 5-hydroxymethyl-C (5hmC), which can be deaminated by AID/APOBECs to 5-hydroxymethyl-U (5hmU) and replaced by C through BER. 5hmC can be further oxidized by TETs proteins to 5-formyl-C (5fC) and 5-carboxyl-C (5caC), leading to activation of BER to restore C. Finally, another potential mechanism of active DNA demethylation would involve 5mC deamination to T and TET-mediated oxidation of T to 5hmU, which would be replaced by C through BER, although this model has not been proven as yet.

In order to understand the link between deaminase activity of AID and DNA demethylation, we will review the current state of knowledge of the mechanisms involved in active DNA demethylation. Flowering plants possess a set of DNA glycosylases (DEMETER, ROS1, DML2, and DML3) capable of recognizing and removing 5mC in a discrete number of loci, such as imprinted genes and silenced transgenes (79–81). Due to the lack of homologs of 5mC DNA glycosylases in vertebrates, other mechanisms of DNA demethylation have been proposed in mammals. It is well established that the BER machinery, which restores nucleotide lesions originated after base deamination, alkylation, or oxidation (82), is involved in the process of active DNA demethylation (83, 84). The most frequent lesion in DNA is uracil, which is removed by members of the UDG family: UNG, thymine DNA glycosylase (TDG), single-strand-selective monofunctional uracil-DNA glycosylase 1 (SMUG1), and MBD4 (85). The first model for 5mC demethylation was described in zebrafish embryos and proposed that deamination of 5mC by AID generated a T:G mismatch that was excised by MBD4, with the cooperation of GADD45, and finally repaired to restore C (45). The formation of a complex containing AID, GADD45, and a DNA glycosylase was also observed in an independent study using HEK293 cells, although the authors identified TDG as the glycosylase of the BER machinery involved in demethylation (86). The discovery of the base 5-hydroxymethyl-2′-deoxycytidine (5hmC) (87) as a result of the 5mC oxidation by proteins of the (ten-eleven translocation) TET family (88) enabled the identification of other possible mechanisms of active DNA demethylation (89). One report proposed that the conversion of 5mC to 5hmC by TET1 initiated an oxidative-deamination process mediated by the coordinated action of AID/APOBEC proteins and BER pathway, which led to DNA demethylation (90). 5hmC deamination by AID generated 5hmU, which was excised and repaired by the BER machinery. The specific 5hmU DNA glycosylases were not identified, but subsequent experiments with TDG^−/−^ mice suggested that TDG was the DNA glycosylase responsible for the excision of 5hmU (or T) after deamination by AID of 5hmC (or C) (86). This model of active DNA demethylation involved the deamination of 5hmC to 5hmU by AID/APOBEC proteins, a step that was questioned later on due to the voluminous size of the hydroxymethyl group (91). The biochemical analysis of the AID enzymatic activity using a ssDNA oligonucleotide deamination assay (ODA) (27) indicated that AID could deaminate C and 5mC, but was unable to remove the side chain at C5 position from 5hmC due to the size of the hydroxyl group (91). In line with these results, Nabel et al. demonstrated that the deamination activity of AID diminished with the increasing size of the substituent at five position of C due to steric hindrance (47). The rate of 5mC deamination was only 10% relative to unmethylated-C, and no activity was detected on 5hmC *in vitro*. Therefore, if *in vitro* findings by Nabel et al. also apply *in vivo*, the role of AID in direct demethylation of 5mC and 5hmC may be limited. As an alternative, Petersen-Mahrt’s group has recently proposed that methylated-Cs do not have to be directly targeted by AID to be demethylated (92). Using an *in vitro* resolution (IVR) assay consisting of a methylated plasmid containing GAL4 DNA-binding sites and a GAL4-AID fusion protein combined with *xenopus laevis* egg extracts-containing molecules of the BER machinery, this study provides evidence for the activation of different DNA repair pathways after AID-mediated deamination. The short-patch-BER machinery restores only one base, whereas the processive DNA polymerase pathways (long-patch-BER or MMR) incorporate multiple nucleotides during the repair process (82, 93, 94). In that last scenario, the deamination of C (or 5mC) to U (or T) by AID would promote the activation of a processive DNA polymerase, which would introduce from 2 to 12 nt in the case of the LP-BER (93), or up to 2 kb of ssDNA if the MMR pathway is triggered (95). As a result, there would be extensive demethylation along the section of DNA around the initial lesion and 5hmC demethylation would occur independently of targeted AID deamination (92). However, the physiological relevance of this model still needs to be confirmed. All these proposed mechanisms of AID-mediated demethylation rely on the lesion-induced activation of the DNA repair pathways. In all scenarios, the deamination activity of AID introduces a modified base that is targeted by the repair machinery. SHM results from recruitment of the error-prone short-patch BER-especially in Ig genes, leading to a single nucleotide substitution, and a loss of a “methylatable” C. The active and direct loss of methylation is possible if AID deaminates 5mC to T. On the other hand, if LP-BER or MMR are involved in repair, the outcome is more marked demethylation that extends beyond the original single nucleotide lesion (96). It remains unclear how AID is recruited to its DNA targets. SHM is linked to transcription: AID requires ssDNA to initiate the deamination of IgV regions (97). Regarding AID partners, it has been demonstrated that stalled Pol II, Spt5, and RPA are necessary for recruitment of AID to Ig and non-Ig targets (98, 99). Duke et al. delineated that a combination of E-box with YY1 and C/EBP-β binding sites targets AID in B cells (100). However, it is not known if demethylation and SHM targets are always the same. Hypothetically, the demethylation targets may exceed the numbers of SHM targets due to successful repair, which would leave the AID target site as mutation-free but demethylated. Also, hypomethylated areas are located in introns, intergenic regions and repeat elements rather than promoters, suggesting a different targeting mechanism (46). In contrast, other studies have proposed that active DNA demethylation does not involve AID/APOBECs-mediated deamination, but it occurs through the different intermediates generated after the successive oxidation of 5hmC by TET proteins: 5-formyl-C (5fC) and 5-carboxyl-C (5caC), which are excised by TDG and replaced by unmodified cytosine (101, 102). Finally, it has been demonstrated recently that TETs are able to oxidize T to 5hmU in mouse ESCs (103). This finding unveils another possible mechanism of demethylation consisting of AID-mediated deamination of mC to T followed by conversion of T to 5hmU by TET proteins. Based on the previously mentioned results, it seems reasonable to conclude that both AID-dependent and TET-dependent mechanisms participate in the active demethylation of the genome (Figure 1). To what extent they are interconnected or which one is preferentially activated depending on the cell type needs to be further investigated.

## DNA Hypomethylation and Cancer

The question of AID activity, its possible link to DNA hypomethylation and predisposition to cancer is a fundamental one. At the moment, only a tenuous link exists between AID-induced SHM and DNA hypomethylation in B cells and B cell lymphomas. There is a large body of evidence though linking hypomethylation to cancer (70, 104, 105). Many subtypes of lymphomas reveal genome-wide hypomethylation. Wahlfors et al. made an early observation that chronic lymphocytic leukemia (CLL) genome undergoes global loss of methylation using digestion of genomic DNA with isoschizomer enzymes *Hpa*II and *Msp*I, followed by validation using HPLC (104). More recently, these findings were confirmed by whole methylome sequencing studies, which revealed that aberrant hypomethylation was centered in repetitive sequences, like ALU and LINES and were particularly pronounced in CLL with TP53 mutations (106). A subset of DLBCLs also revealed loss of DNA methylation, as demonstrated by Chambwe et al. (107). Methylome interrogation in the follicular lymphoma (FL) cell line RL and in CD19^+^ B cells using 454 sequencing technology (108) revealed hypermethylation in the promoters, but hypomethylation in intra- and intergenic areas of the genome.

DNA methylation patterns in all tissues and cell types are a result of two main forces: deterministic patterning and stochastic changes [reviewed in Ref. (109)]. Deterministic changes in DNA methylation reflect the tissue-specific forces, which are dependent on transcription factors and epigenetic factors that reflect the tissue type and determine cellular identity. On the other hand, the stochastic changes reflect the cell-to-cell variability, with individual cells at the same tissue and differentiation stage displaying epigenetic heterogeneity. There are several possible sources of stochastic variability: aging, ambient mutagens, oxidative damage, errors, and off-target activity of epigenetic enzymatic factors (110, 111). Several mechanisms have been proposed to contribute to DNA hypomethylation in normal development and disease, which to different degrees can explain deterministic and stochastic DNA hypomethylation. One explanation is a possible reduction of intracellular concentration of DNA methyl donor S-adenosylmethionine (AdoMet) or an increased concentration of the product of the reaction and its inhibitor S-Adenosylhomocysteine (AdoHcy) (112). Methyl-deficient diet has been shown to lead to AdoMet deficiency in a mouse model (113), but has not been proven to contribute to carcinogenesis in humans. Another logical explanation for DNA hypomethylation would be the decreased activity of DNMTs or increased activity of demethylases. Published observations in tumors and normal development suggest that hypomethylation does not correlate with decreased expression of DNMTs or their mutations (114–116). On the other hand, active demethylation can take place either by the action of yet unidentified demethylases or through DNA repair process. Enzymatic demethylation is not favored because of the stability of the chemical bond. Ramchandani et al. reported the biochemical purification of a DNA demethylase from tumor cells (117). The same group demonstrated that the rate limiting step in the reaction is initiation of demethylation, which is sequence specific and progresses in a processive manner sliding along the DNA and demethylating CpGs in cis position (118). MBD2 was demonstrated to possess demethylase activity in biochemical *in vitro* experiments and in solid cancers, but its function as demethylase remains controversial (119, 120). Another possibility is modification of DNA nucleotides by deaminases resulting in eventual excision and repair of that nucleotide and loss of methylation. AID is the key candidate for this mechanism of demethylation, as discussed before. AID is known to contribute to chromosomal instability and induce chromosomal translocations by inducing DSBs (see below). B lymphocytes from IgκAID-transgenic mice showed increased number of chromosomal translocations (6% of cells) and increased frequency of DNA breaks (8% of cells) (121). The contribution of hypomethylation to AID-dependent genomic instability has not been addressed and warrants further investigation.

The biological effect of DNA hypomethylation is its contribution to reactivation of transposable elements, activation of oncogenes, and increased chromosomal fragility, as demonstrated elegantly by Gaudet et al. in experiments with hypomorphic DNMT1 mice (122, 123). Mice with low DNMT1 expression at 10% of wild type level demonstrated marked reduction in genome-wide DNA methylation and revealed significant increase in genomic instability and activation of proto-oncogenes, like *c-Myc* (124). The maintenance of methylation in mammalian cells is accomplished by DNMTs, particularly DNMT1 and its complex with PCNA and UHFR1. Disruption of this complex results in global hypomethylation and serves as a biomarker of poor prognosis in GBM tumors (125).

## Role of AID in Lymphomagenesis

AID is expressed in half of Burkitt lymphomas (BLs), 30% DLBCLs and 25% FLs, and is absent in B cell precursor lymphoblastic leukemia and mantle cell lymphomas (126, 127). Due to its DNA mutator capacity, AID activity represents a potential risk for genomic instability. It is clear that AID can introduce point mutations in Ig and non-Ig genes and also induce chromosome translocations involving oncogenes, which contribute to the development of B cell neoplasms (128). Pasqualucci et al. found that proto-oncogenes such as *PIM1, MYC, RHOH*/*TTF*, and *PAX5* were aberrantly mutated in more than 50% of the DLBCLs cases analyzed, probably due to failure of the SHM pathway (129). In addition, chromosomal translocations are frequent in lymphomas and myelomas, including DLBCLs, BLs, multiple myeloma, and mouse plasmacytoma (130, 131). Specifically, translocations between *c-MYC* and the Ig C_H_ genes (*c-MYC/IgH*) are a hallmark of BLs and were demonstrated to be AID-dependent, since AID-deficiency eliminated the presence of canonical *cMyc* translocations in BCL6 or Bcl-xL transgenic mice (132, 133), although the frequency of DSBs was lower in *c-Myc* than in *IgH* (76). Moreover, mutations in the anti-apoptotic protein *BCL2* are frequent in DLBCLs and are enriched in the AID-binding motif WRCY (134).

Multiple mouse models have been established to study lymphomagenesis *in vivo*, and more specifically to clarify the role of AID in the induction and development of B cell neoplasms. Experiments with IμHABCL6 transgenic mice, which develop lymphomas due to the deregulated expression of the transcriptional repressor BCL6 under the control of the IgH Iμ promoter (135), demonstrated that AID had a role in lymphomagenesis (136). AID-deficiency in IμHABCL6 background prevented the formation of GC-derived lymphomas. This effect was not observed when crossing *Aicda*^−^*^/^*^−^mice with other lymphoma-prone mouse models such as λMYC mice, which develop pre-GC-derived lymphomas, or λMYC/IμHABCL6 mice, characterized by the formation of post-GC-derived malignancies. The same report showed that *ex vivo* stimulated B cells from IμHABCL6 mice presented a high number of *c-Myc/IgH* translocations, which were not present in activated IμHABCL6/*Aicda*^−^*^/^*^−^cells. The authors proposed that AID-mediated translocations contributed to lymphoma formation (136). Another study reported that AID activity determined the frequency of lymphocytes with *c-Myc/IgH* translocations, influencing the incidence of B cell tumor development in a mouse model of plasmacytoma (137). An independent group demonstrated the link between AID-mediated chromosome translocations and mature B cell lymphomas using another transgenic mouse strain, which expressed AID under the control regulatory elements of the light chain Igκ (121). In this experimental design, deregulated AID expression generated DSBs in the genome of B cells, inducing *c-Myc/IgH* chromosome translocations. Nonetheless, AID overexpression was not sufficient to drive B cell lymphomagenesis, requiring the concomitant loss of the tumor suppressor p53 in that model (121). The generation of a transgenic mouse strain with sporadic *c-Myc* activation in GCBs (Vk*MYC), which led to a multiple myeloma phenotype, indicated that AID-mediated SHM was required for the aberrant *c-Myc* expression and the subsequent plasma cells expansion (138). Constitutive expression of AID using a transgenic mouse model, which expresses AID under the control of the ubiquitous CAG promoter, led to T cell lymphomas containing no translocations but abundant point mutations in *c-Myc* and the *TCR* genes (139). Bone marrow transplantation experiments with AID-transduced cells also resulted in the development of T-lymphomas, with frequent point mutations in *Notch1, PTEN*, and *c-Myc*. Noteworthy, some of the mice presented B-lymphomas after transplantation. *Pax5* and *Ebf1* were mutated in these B cell-derived malignancies and no chromosome translocations were found (140). The aforementioned mouse models proposed different mechanisms of AID-mediated genomic instability, focused on the analysis of somatic mutations or translocations involving proto-oncogenes generated as a consequence of the deaminase activity of AID. Intriguingly, the role of AID activity in the epigenetic stability of the genome and its implication in lymphomagenesis remain largely unexplored. De et al. (141) analyzed the DNA methylation patterning in three subtypes of primary lymphomas: FL, GCB–DLBCL, and ABC–DLBCL (142), which differs in their level of aggressiveness (FL < GCB–DLBCL < ABC–DLBCL). This study demonstrated that normal GCBs presented higher level of epigenetic heterogeneity than NBs, and also that inter-sample and intra-sample methylation heterogeneity increased with lymphoma aggressiveness, correlating with adverse outcome (141). Trying to discover the cause for the abnormal DNA methylation patterns in B cell lymphomas, the authors found that the promoters of the targets of *BCL6* and *EZH2* showed an aberrant hypermethylated status compared to normal B cells. On the contrary, AID target genes presented an abnormal promoter hypomethylation. In addition, the expression level of AID was significantly correlated with genome-wide aberrant hypomethylation (141). This result, along with the established role of AID in active DNA demethylation during normal development and also with the significantly higher hypomethylation in GCBs compared to NBs in regions enriched for the putative AID-binding site RGYW, suggests an epigenetic role for AID during GC transit of normal B cells and in GC-derived lymphomagenesis. Such a role needs to be formally proven and will have great implications on our understanding of B cell biology.

## Author Contributions

Pilar M. Dominguez and Rita Shaknovich conceptualized and wrote the manuscript.

## Conflict of Interest Statement

The authors declare that the research was conducted in the absence of any commercial or financial relationships that could be construed as a potential conflict of interest.

## Abbreviations

BCR, B cell receptor; BER, base-excision repair; BL, Burkitt lymphoma; CSR, class switch recombination; DLBCL, diffuse large B cell lymphoma; DNMTs, DNA methyltransferases; DSBs, double strand DNA breaks; EMT, epithelial–mesenchymal transition; ESC, embryonic stem cells; FL, follicular lymphoma; GCBs, germinal center B cells; Ig, immunoglobulin; iPSCs, induced pluripotent stem cells; MMR, mismatch repair; NBs, naïve B cells; PGCs, primordial germ cells; SHM, somatic hypermutation; TLR, toll-like receptor.
